# From Stress to Burnout: Exploring the Protective and Predictive Factors for Nurses’ Well-Being

**DOI:** 10.3390/healthcare14101423

**Published:** 2026-05-21

**Authors:** Suad Dukhaykh, Shaikhah Bawzeer

**Affiliations:** Management Department, College of Business, King Saud University, Riyadh 11451, Saudi Arabia

**Keywords:** occupational stress, burnout, job satisfaction, self-efficacy, nurses

## Abstract

**Background/Objectives:** Occupational stress is a prevalent issue in healthcare settings, particularly among nurses, and is often associated with increased levels of burnout and reduced well-being. This study aims to examine the relationship between occupational stress and burnout among nurses, with a particular focus on the mediating role of job satisfaction and the moderating role of self-efficacy. **Methods:** A quantitative research design was employed using data collected from 245 nurses in Saudi Arabia through a bilingual survey instrument incorporating validated psychological measures. Statistical analyses were conducted to test the direct, mediating, and moderating relationships among the study variables. **Results:** The findings indicate that occupational stress is positively associated with burnout and negatively related to job satisfaction. Job satisfaction was found to partially mediate the relationship between stress and burnout, suggesting that reduced job satisfaction serves as a key mechanism through which stress contributes to burnout. In contrast, self-efficacy did not demonstrate a significant moderating effect in this relationship. **Conclusions:** This study contributes to the occupational health literature by highlighting the critical role of job satisfaction in mitigating the adverse effects of stress on burnout among nurses. The findings offer practical implications for healthcare leaders and policymakers seeking to design targeted interventions aimed at enhancing job satisfaction, reducing burnout, and improving nurses’ overall well-being.

## 1. Introduction

Nurses are indispensable in any healthcare system as they help with caring for patients, managing illnesses, and attending to emergencies. Their role requires them to have extensive medical knowledge, exceptional communication skills, and remarkable psychological stamina. Nevertheless, their occupation’s rigorous and exhausting schedule coupled with relentless work results in chronic occupational stress, which could have significant psychological, emotional, and career-related consequences [1]. According to the World Health Organization, occupational stress stemming from heavy workloads, rotating shifts, and emotionally demanding interactions is a major contributor to mental health issues among healthcare professionals [2]. Within the nursing profession, occupational stress is intensified by insufficient staffing levels, disproportionate nurse-to-patient ratios, administrative burdens, and the emotional toll associated with patient care outcomes. Chronic occupational stressors not only impact the mental health of nurses, but also affects patient safety, their job performance, and the overall efficacy of healthcare in a direct manner [3]. Nurses, psychologically strained over a prolonged duration, typically experience exhaustion, stress, and diminished professional satisfaction; if this persists, burnout is likely to occur in the future [4]. Burnout stands out as one of the most serious psychological outcomes resulting from occupational stress [5,6].

Research on occupational stress and burnout has consistently demonstrated a definitive correlation. Burnout impairs morale at work, increases turnover rates, and worsens the quality of care provided to patients, consequently carrying its consequences beyond individual nurses. Higher absence rates, more medical errors, and less efficient healthcare services are all contributing to burnout. Nurses who experience burnout out are more likely to make substantial errors that place patients at risk, compromising their well-being [6]. In view of that, healthcare organizations that seek to improve patient care and employee wellness ought to consider addressing burnout their primary priority [1]. While some studies have linked self-efficacy, which indicates individual’s belief in their capability to accomplish a specific task or achieve a desired outcome, to a lower probability of burnout, the results remain inconclusive in relation to various healthcare settings [7,8]. Stronger and better emotional regulation has been linked to a higher degree of self-efficacy, according to some studies. Nevertheless, additional research suggests that when there are numerous patients in need of intervention and not enough staff, self-efficacy is inadequate [9,10]. Research has already demonstrated a connection between a lower risk of burnout and job satisfaction. However, it remains unclear if job satisfaction is a universally applicable element or whether its effects vary depending on the working environment, standards, and culture [11,12]. Burnout correlates with an elevated probability of medical errors, decreased efficiency, and higher absenteeism, among other consequences, which compromises the hospital’s efficiency and welfare of patients [13]. Studies reveal that nurses who suffer from burnout are nearly three times more likely to commit clinical errors, which may endanger patient safety, extend hospital stays, or lead to fatalities [14]. This paper analyzes the synergistic effect of job satisfaction and self-efficacy. This will aid researchers, lawmakers, and administrators in devising customized solutions for alleviating burnout and improving overall job conditions. The study’s findings will specifically aid nursing educators, hospital managers, and legislators in ameliorating nurses’ working circumstances, instituting stress reduction strategies, and boosting job satisfaction across nurses. Additionally, this study has broader implications for global healthcare systems. Given the worldwide nurse shortage, burnout-related turnover needs to be addressed if we want to maintain a consistent and effective workforce. This study can serve as the foundation for future policy reforms that aim to reduce workplace stressors, promote psychological resilience, and increase job satisfaction in a variety of healthcare situations [14].

By integrating organizational (job satisfaction) and psychological (self-efficacy) components into a single model, this research seeks to enrich the academic discourse surrounding burnout by offering empirical insights. This builds upon previous studies and introduces a more integrated perspective for comprehending and addressing burnout among nurses.

## 2. Review of the Literature

### 2.1. Occupational Stress Among Nurses

Nurses endure the most stress compared to other professions, and this is due to work-related psychological, emotional, and physical trauma [12]. The intensely dynamic and strained environments in which nurses operate require them to cope with acutely ill patients, multifaceted health crises, moral challenges, and even some clerical work within strict boundaries designed to deliver optimal patient satisfaction. This combination leads to stress becoming, unfortunately, a universal experience for nurses globally, an experience that has negative consequences on their mental and physical well-being if not properly managed [1]. One of the most serious consequences of unmanaged stress is burnout. Burnout is commonly defined as a psychological condition marked by emotional exhaustion, a sense of detachment or depersonalization, and a perceived decline in personal accomplishment. Initially defined by Freudenberger in 1974, burnout has since been understood to be a significant risk in the healthcare sector, especially nursing, because of the long-term contact with emotionally painful situations. Burnout has been officially recognized by the World Health Organization (WHO) as an occupational condition, given its clear impact on healthcare workers’ psychological well-being, job performance, and overall health [2]. Evidence suggests that the levels of burnout in nursing are closely related to issues of safety, mistakes in medicine, and high attrition rates, which makes it an issue of concern for hospital managers and policymakers. With the rise in burnout among healthcare professionals, researchers are looking for psychosocial buffers that can help alleviate occupational stress and prevent progression of burnout. Self-efficacy, the belief of performing competently in job-related tasks, has been shown to moderate the impact of stress, assisting the nurses’ ability to deal with strenuous situations and minimizing chances of burnout [8]. Moreover, job satisfaction has an impact on whether occupational stress results in burnout or can be alleviated due to positive encounters at work [12]. Understanding these relationships is essential for designing effective, evidence-based strategies that support nurses’ psychological health and promote their continued presence within the healthcare workforce. Burnout has been identified as one of the worst mental effects of long-term professional stress in healthcare workers. It changes slowly over time because of long-term exposure to job expectations that are too much for a person to handle. Researchers have found a few main things that make nursing staff tired: a lot of work, being emotionally worn out from caring for patients, working shifts, and not having enough freedom at work [9]. Nurses employed in high-stress environments, such as emergency departments and intensive care units, are significantly more susceptible to experiencing burnout. In fact, a study shows that over 70% of ICU nurses experience moderate to severe burnout symptoms [15]. A study on occupational burnout by Maslach and Leiter (2016) [1] showed that it is not just an issue for one person, but a problem for the whole company. Burnout at work is made worse by things like not enough staff, bad leadership, and few chances to move up in the company. This makes it hard for nurses to stay involved in their jobs [1]. Similar trends have been found in Saudi Arabia, Egypt, and Jordan. They show that nurses in hospitals with few resources are more likely to become burned out because they must deal with more patients per nurse and too many routine tasks [16,17,18]. In addition, enduring stress leads to physiological reactions that induce stress in the body, which produces hormonal distresses (such as raised cortisol levels) that worsen burnout symptoms. As a result of severe burnout, nurses often present with psychosomatic complaints which may include insomnia, chronic fatigue, anxiety, and depressive symptoms, thus placing the patients at risk of compromised care [19]. Considering the primary responsibilities of nurses in the health sector, it goes without saying that burn out causes major issues, which need immediate attention both at individual and organizational levels.

Likewise, Egypt and Jordan struggle with limited healthcare resources, making it difficult to address nurse burnout and occupational stress. Contributing factors include prolonged duty hours, persistent staffing shortages, and insufficient financial support. Because of limited resources available to public healthcare institutions, patients are overloaded, nurses are underpaid, and there is inadequate professional development available—and all that leads to high stress levels in Egyptian nurses [17]. A majority of nurses in Cairo’s general hospitals report experiencing burnout, with many identifying emotional exhaustion as the main contributing factor to their discontent with the nursing field and their decision to consider leaving the profession [20].

Research in the Egyptian healthcare context has explored the link between self-efficacy and stress, revealing that nurses with high self-efficacy tend to engage more effectively in their professional roles and are less likely to experience burnout [21]. Nurses with high job satisfaction also reported lower burnout and studies show that having supportive management teams that enable career advancement programs greatly improves burnout rates, even in high-demand environments [22]. Evidence from Jordan also highlights occupational stress as a significant contributor to burnout among nurses, especially within public sector healthcare services where funding and staffing remains a problem. A study carried out in Amman governmental hospitals revealed that stress arising from a high patient workload, administrative burden, and lack of career advancement was greatly associated with emotional exhaustion and depersonalization of self-nursing [23]. But, this time, self-efficacy emerged again as an important construct; findings suggested that nurses who had confidence in their professional competencies and their ability to solve problems reported low levels of burnout even when they encountered stressors at the workplace [24].

The phenomenon of work stress and burnout among Saudi nursing staff has attracted much concern due to the increased healthcare demand and several other pressures on the nursing workforce. Saudi Arabia has witnessed a broad development of healthcare facilities because of Vision 2030, leading to a greater dependence on both Saudi and foreign nurses. Yet, research shows that, perhaps due to the fast pace of hospital work, high nurse-to-patient ratios and the nature of caring for patients’ burnouts has become the most significant problem in Saudi nursing. According to a new study carried out in Riyadh’s public hospitals, 63% of nurses reported suffering from moderate to severe burnout, with emotional exhaustion being the most common symptom suffered [25]. The study pointed out disproportional work overloads, insufficient attention from managers, and shift work rotation as the most contributive factors to stress and burnout. Fascinatingly, studies on Saudi Arabia have also looked at how self-efficacy moderates the work enticements. Higher self-efficacy Saudi nurses were notably less likely to feel emotional tiredness and depersonalization according to a study looking at the link between occupational stress and burnout [26]. This implies that shielding nurses from the detrimental consequences of work stress depends much on psychological resilience. Moreover, the study underlined that those nurses who had continuous professional development and mentoring showed better degrees of self-efficacy, thereby underlining the need for occupational development initiatives regarding reducing burnout. Researchers have examined job satisfaction as a mediating variable within nursing practice in Saudi Arabia, demonstrating that nurses who are gratified with their workplace and compensatory structures and have opportunities for career advancement experience lower levels of burnout even in stressful situations. In a regionwide study conducted in public and private hospitals in Dammam, it was found that nurses with higher job satisfaction showed greater organizational commitment and professional resilience, thereby diminishing their vulnerability to burnout [27]. These results emphasize the need of workplace reforms directed at bolstering salary levels, nurse–patient ratios, and managerial assistance in Saudi hospitals.

### 2.2. Identification and Analysis of Burnout in Nurses

Burnout develops as a psychological condition resulting from ongoing exposure to intense stressors that are not adequately addressed over an extended duration. It often features emotional fatigue, depersonalization, and loss of self-fulfillment. “Psychological” burnout, as defined by Maslach and Leiter in 2016, is marked by feeling worn out and emotionally detached while being less competent in professional duties [1]. According to the WHO, burnout is not classified as a medical condition but rather as a consequence of ongoing occupational stress that has not been effectively managed. It reflects a state in which individuals are unable to achieve sufficient rest due to constant demands that consume nearly all their time, often without meaningful progress on tasks [2]. Out of all groups of healthcare workers, nurses seem to suffer the most since the profession is characterized by having to meet tough job requirements, emotional stress, suffering around, and constantly seeing dead bodies. The issue of burnout in nurses is complex and has implications on personal health, quality of care, and institutions. Nurses’ burnout is linked with many medical errors, low satisfaction, absenteeism, and high turnover that makes the organization ineffective and harms patient safety [28]. The increased prevalence of burnout has called for more scrutiny towards its potential causes and solutions [5]. The Copenhagen Burnout Inventory (CBI), developed by Kristensen et al. (2005) [29], assesses burnout as a state of physical and psychological exhaustion across three dimensions: personal burnout, work-related burnout, and client-related burnout. Personal burnout reflects the overall level of fatigue and exhaustion experienced by individuals, regardless of their occupational context, and represents a general depletion of physical and emotional energy. Work-related burnout refers specifically to exhaustion that individuals attribute to their work, capturing the extent to which job demands contribute to mental and physical strain. Client-related burnout focuses on exhaustion arising from interactions with recipients of one’s work, such as patients, students, or customers, and reflects the emotional burden associated with continuous interpersonal demands. The CBI has been widely utilized in both clinical and research settings due to its strong psychometric properties and its ability to distinguish between different sources of burnout, thereby providing a more nuanced understanding that can inform targeted interventions. The issue of burnout, particularly in people-oriented professions, remains a global concern. A study by Adriaenssens, De Gucht, and Maes indicated that over 50 percent of nurses suffer from moderate to severe burnout symptoms, with those in emergency and critical care reporting the most severe symptoms [30].

Comparable studies from Saudi Arabia reveal that nearly 63 percent of nurses report experiencing burnout, with persistent emotional fatigue emerging as the most prominent manifestation of the condition [31]. These result highlight that while burnout is a common health hazard in the workplace, geriatric healthcare systems seem to have a worse impact. Burnout in nursing is often the result of personal, occupational, and organizational factors. One of the primary causes is excessive workload and job demands. Nurses tend to work long hours, have erratic shift patterns, and are assigned large numbers of patients to tend to, leading to cumulative stress and exhaustion [14]. Critical environments such as intensive care units and emergency departments also require nurses to manage very important patients in much shorter time frames, which can lead to even higher levels of stress [30]. Burnout can also be caused by emotional strain and compassion fatigue. Nurses face extremely emotionally challenging situations, including dealing with patient suffering, facing mortality, and death, which can lead to emotional draining over time. It has been noted that nurses in palliative care and Oncology departments suffer from compassion fatigue, which increases the rates of burnout [32]. Such emotionally challenging work can lead to being mentally fatigued and therefore nurses become less empathetic and engaged at work [28]. Absence of an organization’s assistance is recognized as a further key factor contributing to burnout. Ineffective supervision, lack of hands-on support from managers, and absence of professional growth options tend to depersonalize and disengage nurses from their work. Nurses who view their workplace context as unsupportive are more prone to burnout because they have insufficient means to deal with job stresses. Laschinger, Finegan, and Wilk argued that autonomy at work and the ability to make decisions are critical to nurses’ engagement, and their absence severely undermines professional burnout [33]. In 2017 Leiter and Maslach further emphasized that deprivation of meaningful work engagement plus non-recognition increases the likelihood of depersonalization and emotional exhaustion in nurses [34]. Another prominent contributor to the phenomenon of burnout among nurses is imbalance between work and leisure. The nature of nursing is so demanding that it leads to conflicts of work and family responsibilities that adversely affect relationships and general health. Nurses experiencing insomnia, sedentary lifestyle, and insufficient time off report high levels of burnout [35]. Khamisa et al. had shown evidence that stressed nurses devoid of time for personal care and family activities suffer more due to occupational burnout, which makes the situation worse [36].

Nurse burnout is a high-priority occupational health issue, which also affects the outcome of the healthcare professionals and patients alike. Due to negative impacts on patient safety, job satisfaction, and retention of the workforce, burnout is an issue that needs attention at systemic and personal levels. Some actions associated with minute alterations in organizational structure, such as reducing the workload, increasing support from the management, as well as promoting self-efficacy and job satisfaction among nurses, are essential for decreasing the chances of burnout [37]. For designing effective strategies supporting nurse welfare and a robust healthcare system, it is important to comprehend the reasons and effects of burnout. In developing Middle Eastern countries, burnout among nurses is a growing problem and rigid working conditions, lack of resources, high number of patients, and other factors cause considerable work-related strain. Studies conducted in Egypt, Jordan, UAE, Lebanon, and Iraq highlight that burnout is one of the most challenging problems among nursing staff, especially in the public sector hospitals where there is an insufficient number of medical workers and basic facilities, and equipment are also lacking, and this imposes more pressure on healthcare professionals. In 2004 over 60 percent of nurses in public hospitals in Jordan reported feeling emotionally exhausted alongside experiencing moderate to severe burnout. Nurses in emergency and critical care units experienced the highest levels of burnout due to long working hours, high patient acuity, and poor managerial support [38]. Egyptian research illustrates that more than 70 percent of general hospital nurses experience burnout because of low pay, job insecurity, and poor career advancement opportunities [20]. In the UAE, burnout among nurses has been ascribed to the large number of expatriate nursing personnel, who frequently encounter cultural and linguistic challenges, as well as ambiguities regarding work contract legalities.

In a study on burnout conducted among nurses in Dubai’s healthcare sector, job satisfaction was identified as a key factor that clearly reduced the negative impact of workplace stress. On the other hand, burnout was much higher among nurses who had little to no autonomy over their workdays and schedule [39]. Burnout among nurses in Lebanon is often associated with economic and political instability, as well as chronic underfunding of healthcare institutions and increasing workload demands on medical personnel. A significant number of nurses from Lebanon reported feeling emotionally distanced from their job owing to constant exposure to humanitarian crises coupled with a lack of medical resources [40]. As for Iraq, with the least stable countries, the healthcare system has some of the highest burnout levels in the region, especially among nurses working in conflict-ridden areas. A previous study in Babylon found that 57.8% of the nurses experienced moderate burnout [41]. In Saudi Arabia, the issue of nurse burnout is one of the concerns for healthcare practitioners and managers because of the underlying dependency of the healthcare system on foreign nurses, as well as the country’s increasing dependence on this sector. Current literature suggests that like most of the developing countries, Saudi Arabia suffers from burnout among nurses, which is equal to or higher than the global average and is the worst among public sector nurses who are overworked with long shifts, insufficient staff, and an excessive number of patients [31]. In one cross-sectional analysis of public hospitals in Riyadh, 63% of the nurses studied said they suffered burnout symptoms ranging from moderate to severe, with the most prevalent symptom being emotional exhaustion [25]. Work overload, lack of organizational support, and inflexible work schedule rotations were identified as the most important causes of burnout. Overwhelming workload, poor managerial assistance, and shift work rotation were found to significantly contribute to burnout. The same findings have been observed in Jeddah’s tertiary care hospitals, where nurses assigned to intensive care and emergency settings reported heightened levels of work-related stress [16]. Roughly 60% of the nursing force in Saudi Arabia comprises expatriate nurses, and this demographic makeup poses a challenge. Nurses that are expatriates tend to experience additional stressors such as cultural adjustment, job insecurity, and language barriers [11]. Expatriate burnout in Saudi Arabia has also been attributed to inequality in the private and public health systems within the country. Nurses in the private sector are more satisfied with their jobs and report lower levels of burnout because of better salary packages, working conditions, and greater flexibility in scheduling. However, nurses in government hospitals face a greater number of patients, higher levels of bureaucracy, and lower rates of career progression, which contributes to greater levels of burnout [16]. The government of Saudi Arabia noticed burnout as an important issue and took initiatives to improve the well-being of nurses. Programs like the Vision 2030 Healthcare Transformation Program try to alleviate somatic nurses’ burnout by improving professional training, working environment, and nurse-to-patient ratios [25].

### 2.3. Self-Efficacy

Self-efficacy, which reflects an individual’s belief in their capacity to carry out responsibilities and cope with challenges in the work environment, is regarded as a vital element of psychological resilience in the nursing profession [8]. In Saudi Arabia, nurses who held strong self-efficacy beliefs were found to be emotionally exhausted and experiencing depersonalization, which was caused by the high-pressure hospital environment [26]. At the same time, research in Egypt and Jordan supports the concept of self-efficacy as a mediator of burnout on nurses’ psychological well-being when work demands are high [21,24]. Considering the defensive function of self-efficacy, moderators must aim at increasing the self-confidence of nurses regarding their scope of practice. 

### 2.4. Job Satisfaction

Job satisfaction has an important mediating function on how occupational stress results in burnout and whether there are favorable psychosocial workplace conditions that can moderate the impact of such stressors [36]. On the other hand, when nurses are not satisfied with their job, there is a greater impact of stress leading to increased emotional fatigue and higher nurse attrition. A number of studies corroborate that job satisfaction, either fully or partially, serves as a mediator between occupational stress and burnout. In a study performed in Saudi hospitals, nurses who reported high job satisfaction had lower burnout rates even when they experienced high stress levels [31]. Researchers in Egypt and Jordan found that nurses with career advancement opportunities, positive senior managerial support, and work–life balance experienced lower levels of stress-related burnout [22,24]. Existing literature on self-efficacy, burnout, job satisfaction, and occupational stress reveals that there is still critical information missing. Closing these gaps is critical for formulating and implementing effective interventions that can enhance the psychological resilience and job satisfaction of nurses, which translates into better patient care and retention of the workforce. Minimal Investigation on the Moderating Effect of Self-Efficacy. However, the lack of research on the effect of self-efficacy as a buffer for stress and burnout for nurses is surprising, considering how self-efficacy has been studied extensively in general occupational psychology [7]. Studies have indicated that higher levels of self-efficacy are associated with reduced burnout among nurses. However, there is a scarcity of research exploring self-efficacy as a moderating variable in the stress–burnout relationship across diverse healthcare environments. Almost all studies were conducted on self-efficacy in coping faculties instead of focusing on more sophisticated interactions between self-efficacy and workplace stressors such as emotional labor, ethical issues, and high mortality rates of patients. In addition, this gap in the literature is not only limited to the self-efficacy research. Nurses’ burnout training programs remained underdeveloped in Saudi Arabia, Egypt, and Jordan [18,21,26]. There is an urgent need for more experimental or longitudinal studies that examine whether burnout among nurses in these areas can be alleviated by implementing self-efficacy enhancement interventions such as resilience training, mentorship, and cognitive behavioral strategies. Even though it is common knowledge that job satisfaction is an important precursor of burnout, there have been studies failing to find consistent evidence of its mediating effect [36]. Several studies propose that job satisfaction serves as a complete mediator in the relationship between stress and burnout. In Saudi Arabia, for example, nurses employed in government hospitals claim to be less satisfied with their jobs than those working in private healthcare institutions. Nevertheless, the two groups exhibit similar levels of burnout suggesting that other factors may confound the relationship [27]. In addition, cultural and economic differences between various healthcare systems affect job satisfaction further, which renders certain intervention-based strategies ineffective. In the Middle East region, particularly Saudi Arabia, Egypt, and Jordan, research is in a nascent stage. There are specific cultural, economic, and institutional difficulties that these nurses face, which calls for more in-depth grounded research. Consider, for instance, the Saudi economy, which relies heavily on foreign nurses, who, in addition to their work, must deal with stresses from culture shock and language issues [11,25]. These constructs may add to stress or fatigue from work, but the literature does not capture this as well as it should. As in Saudi Arabia, public health systems in Jordan and Egypt are also perpetually underfunded and overcrowded, which creates a different set of stressors for nurses that Western models of burnout have not been able to fully explain [20,23,24]. Subsequent studies should enhance the development of region-specific models that incorporate the sociocultural aspects of stress and burnout as well as the organizational workplace structures and policies of the region. This line of research will enable more precise designs that meet the needs of nurses from the Middle East. Nursing self-efficacy, burnout, and job satisfaction have largely been studied in cross-sectional designs, which means there is only one measurement for each variable over a certain period [32,34]. Furthermore, there is a strong need for experimental research to evaluate the impact of distinct suggests, like training programs for leaders, exercises to foster psychological resilience, and physical changes to the work environment, to create best practice guidelines based on evidence. Through the examination of these research gaps and central issues, this study centers on the knowledge towards the prevention of burnout and proposes measures to mitigate burnout and enhance the psychological wellness and job contentment of nurses. With the escalating worldwide need for health workers, it becomes imperative to protect and strengthen the nursing human resources to provide quality healthcare services to patients sustainably [36,37].

## 3. Hypotheses Development and Research Model

### 3.1. Occupational Stress and Burnout

In 2017, Fumis et al. discussed the distressing consequences of ethical dilemmas regarding burnout within critical care providers and proposed moral distress as a key explanatory factor [42]. In the same way, Browning in 2019 noted that some critical care nurses suffer from excessively elevated levels of burnout in relation to their capacity because of the challenges involved in their profession [42]. Additionally, Moss et al. noted that because of high job expectations, inadequate work–life integration, and lack of mastery over their time, critical care healthcare professionals are prone to burnout [43,44]. Arias-Ulloa et al. suggest that psychological distress correlates with stress and burnout levels among healthcare workers. Considering these results, many researchers have noted the correlation that exists between stress and burnout in nursing [45]. This evidence supports the current hypothesis asserting a direct positive relationship between occupational stress and burnout.

**H1**:  *Occupational stress has a positive impact on burnout.*

### 3.2. Occupational Stress and Job Satisfaction

Frese’s study (1985) highlights the negative relations between stress and job satisfaction, focusing on working conditions such as noise and conflict [46]. Also, Parkes in 1982 found that student nurses in higher stress environments reported lower satisfaction than their peers in lower stress areas [47]. Moreover, a prospective study done in 2023 by Kim and others has shown that sustained work-related stress is one of the primary predictors of nurses’ low job satisfaction and premature withdrawals from the field of nursing [48].

Given the outcomes of prior studies, it is reasonable to infer that considerable evidence supports a negative association between work-related stress and job satisfaction. This forms the rationale behind the second hypothesis, which posits that occupational stress adversely influences the degree of satisfaction individuals derive from their work.

**H2:**  *Occupational stress has a negative impact on job satisfaction.*

### 3.3. Job Satisfaction and Burnout

Feeling satisfied in one’s professional role plays a vital role in reducing the risk of burnout. Nurses who care for patients in environments where they feel appreciated, supported, and actively involved in their duties are statistically less prone to experiencing burnout. In a 2015 systematic review and meta-analysis, Theorell and colleagues concluded that dissatisfaction with work environment significantly contributes to elevated burnout levels among employees [49]. Likewise, in the findings of Häusser et al. concluded that job satisfaction serves a mediating role in the relation between work demands and psychological well-being, thus influencing burnout levels [50]. Another study published by Zhang et al. showed that violence against nurses results in lower levels of satisfaction with one’s job and higher rates of burnout, reinforcing the need for a healthy and dignified work environment [51].

**H3:**  *Job satisfaction has a negative impact on burnout.*

### 3.4. Job Satisfaction as a Mediating Effect

Excessive stress in the working environment can result in lower job satisfaction, which leads to burnout. A study by Häusser and colleagues in 2010 demonstrated that job satisfaction can act as a psychological buffer, reducing the likelihood of experiencing burnout in stressful work settings [52]. At the same time, Theorell and others in 2015 noted that employees in high-satisfaction jobs had reduced rates of burnout even with the presence of occupational stressors [49]. Additional examination of the psychosocial safety climate (PSC) shows that low PSC increases job demands and burnout, and higher PSC improves job satisfaction and decreases chances of burnout [53]. Based on findings from previous research, there is sufficient empirical support affirming our fourth hypothesis, which examines the mediating effect of job satisfaction on the association between occupational stress and burnout.

**H4:**  *Job satisfaction mediates the relationship between occupational stress and burnout.*

### 3.5. Self-Efficacy as a Moderating Effect

Studies have shown that self-efficacy impacts some people positively because they perceive stressors to be less challenging and emotionally distressing. In a cross-sectional study involving 365 nurses, it was discovered that nurses’ self-efficacy moderated occupational stress levels and manifested in mental health issues. Self-efficacious nurses demonstrated lesser stress related to mental health issues, indicative of a protective influence in job satisfaction [54]. A related investigation was carried out by Pisanti et al. in 2015 [55], focusing on occupational coping self-efficacy (OCSE) and its capacity to buffer the psychological consequences—specifically emotional exhaustion and distress—resulting from diminished job control. Their findings support the notion that nurses with high self-efficacy, in turn, are not as adversely affected by work stress [56]. In their assessment of psychiatric nurses during the COVID-19 pandemic, Lim et al. found that burnout played a moderating role in the link between self-efficacy and job performance. The study implies that self-efficacy exerts protective influences on job satisfaction as well, because high-performing nurses who suffered from burnout continued to perform well due to high self-efficacy [9]. Equally, Azemi et al. performed a cross-sectional survey across multiple hospitals in 2022 that showed that self-efficacy led to better mental health outcomes for hospital nurses, and subsequently higher job satisfaction [10]. These findings are consistent with those reported by Cabrera-Aguilar et al., who identified self-efficacy as a moderating factor in the connection between workplace stress and levels of work engagement [57]. The studies revealed that nurses who possessed strong self-efficacy maintained active involvement in their work responsibilities. Jex and Bliese in 1999 found that self-efficacy played a moderating role in the relationship between workplace stressors and job satisfaction.

**H5:**  *Self-efficacy moderates the relationship between occupational stress and job satisfaction.*

### 3.6. Research Model

**Figure 1:**
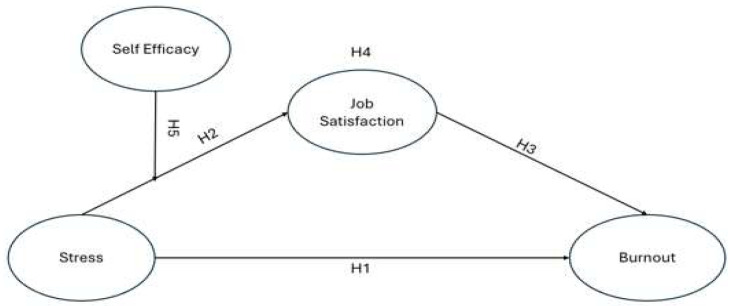
The research model.

Following the discourse of the research hypotheses, the proposed research model showcased in Figure 1 can be illustrated.

## 4. Research Methodology

### 4.1. Sample and Data Collection

The target of this research is the nursing staff working at King Khalid University Hospital, a major academic healthcare institution located in Riyadh, Saudi Arabia. A non-probability convenience sampling technique was employed, as it enabled the researchers to collect data efficiently from accessible participants within the hospital setting. The survey was administered electronically through Google Forms. The link was initially distributed to nursing department heads, who were then asked to share it with their respective teams. To enhance the response rate, a follow-up reminder was sent three days after the initial distribution. Participation in the study was entirely voluntary, anonymous, and confidential, and informed consent was obtained at the beginning of the survey. The study sample consisted of a diverse group of participants (N = 245) from various age groups, genders, nationalities, educational backgrounds, levels of experience, and hospital departments. In terms of age, the largest proportion of participants were between 25 and 35 years old (40.8%), followed by those aged 36 to 44 years (29%), under 25 years (22%), and 45 years and above (8.2%). Regarding gender, 67.8% were female and 32.2% were male, indicating a female-dominated sample. In terms of nationality, 62% were non-Saudi, while 38% were Saudi nationals. For educational attainment, the majority held a diploma (57.6%), followed by a bachelor’s degree (31%) and a master’s degree (11.4%). When examining professional experience, 35.9% had more than 15 years of experience, 31.8% had 9–15 years, 22% had 0–3 years, and 10.2% had 4–8 years of experience.

Participants were employed across various hospital departments, with the highest representation from the Ward—Surgery (22%), followed by ER (18.8%), Internal Medicine (16.7%), and Operating Room (8.6%). Smaller groups worked in departments such as Cardiac Catheterization (6.1%), OPD (6.1%), ICU (4.9%), Orthopedics and Pediatrics Wards (each 4.5%), and Oncology (2.9%). The Cardiology department had the smallest representation at 2%. Table 1 summarizes the demographic attributes of the 245 respondents.

### 4.2. Measures

This study utilized four standardized and validated instruments to measure the primary constructs: perceived stress, self-efficacy, career satisfaction, and burnout. The bilingual format of the questionnaire (Arabic and English) has facilitated communication with the respondents. An officially validated Arabic translation of three of the scales was available, while the fourth was translated utilizing a forward and backward translation method to assure conceptual equivalence and comprehension [58].

#### 4.2.1. Perceived Stress Scale (PSS)

Cohen, Kamarck, and Mermelstein (1983) developed the PSS, which is one of the most popular psychological tools for measuring perceived stress [59]. The 10-item version of the scale was used for this study; the items are rated on a 5-point Likert scale of frequency from 0 (never) to 4 (very often). Sample item: “In the last month, how often have you been upset because of something that happened unexpectedly?” The validated Arabic translation of the scale was utilized [60].

#### 4.2.2. General Self-Efficacy Scale

This 10-item scale was developed by Schwarzer and Jerusalem (1995) to assess a general sense of perceived self-efficacy [61]. It evaluates an individual’s belief in their ability to handle a variety of difficult demands in life. Items are scored on a 4-point Likert scale ranging from 1 (not at all true) to 4 (exactly true). Sample item: “I can always manage to solve difficult problems if I try hard enough.” A validated Arabic translation was used in the current study [62].

#### 4.2.3. Career Satisfaction Scale

Originally developed by Greenhaus, Parasuraman, and Wormley (1990), this 5-item scale assesses an individual’s satisfaction with various aspects of their career progression and success [63]. Items are scored using a 5-point Likert scale from 1 (strongly disagree) to 5 (strongly agree). Sample item: “I am satisfied with the success I have achieved in my career.” An officially published Arabic version of the scale was utilized [64].

#### 4.2.4. Burnout Inventory (CBI)

Originally developed by Kristensen, Borritz, Villadsen, and Christensen (2005) [29], the Copenhagen Burnout Inventory (CBI) is a 19-item scale designed to assess burnout as a state of physical and psychological exhaustion across three dimensions: personal burnout, work-related burnout, and client-related burnout. Items are measured using a 5-point Likert-type scale reflecting either frequency (e.g., from “never” to “always”) or intensity (e.g., from “to a very low degree” to “to a very high degree”), and are subsequently transformed into a 0–100 scoring system, with higher scores indicating higher levels of burnout. A sample item is: “How often do you feel tired?” The scale has demonstrated strong reliability and validity across different occupational contexts. An officially published Arabic version of the scale was utilized [29].

### 4.3. Statistical Analysis

Data was analyzed using SPSS (Statistical Package for the Social Sciences, version 29) software and AMOS (Analysis of Moment Structures, version 29). Descriptive statistical analysis on study variables was performed using SPSS, which included mean, median, standard deviation, and correlation coefficient calculations. With respect to measurement and structural models, confirmatory factor analysis (CFA) was performed together with structural equation modeling (SEM) utilizing AMOS. CFA was implemented to test the validity of the measurement model to confirm that each construct was captured by its indicators, and SEM tested the amount of interrelation between perceived stress, burnout, self-efficacy, career satisfaction, and the hypothesized mediating and moderating effects.

## 5. Results

### 5.1. Data Analysis

Statistical Package for Social Science (SPSS) was used to conduct descriptive data analysis. Confirmatory factor analysis (CFA) was performed with AMOS to estimate the parameters of the model. Finally, the study hypotheses were tested using structural equation modeling (SEM) in AMOS.

#### 5.1.1. Descriptive Analytics

Table 2 illustrates the means, standard deviations, intercorrelations, and reliability values of the hypothesized model.

#### 5.1.2. Measurement Model

Confirmatory factor analysis (CFA) was conducted to validate the hypothesized model concerning the factors and their relationships with the observed variables. The CFA results demonstrated a good fit to the data [65] as indicated in Table 3. Furthermore, the factor loadings were found to be sufficiently high and statistically significant, confirming that the observed variables are strongly associated with their respective latent constructs, as detailed in Table 4.

Additionally, the composite reliability (CR) and average variance extracted (AVE) values exceeded the recommended thresholds of 0.70 and 0.50, respectively, which is essential for establishing the reliability and validity of the measured constructs [66]. According to the guidelines provided by Hair et al., the CR value should be greater than the AVE value. In this analysis, this condition was satisfied, thereby confirming the constructs’ convergent validity.

#### 5.1.3. Hypotheses Testing

Table 5 shows the results of the direct, mediation, and moderation effects of the tested structural model. The results indicate that occupational stress has a positive and significant effect on burnout (β = 0.617, *p* < 0.00). Hence, H1 is supported. In contrast, the results revealed that there is a negative and significant relationship between occupational stress and job satisfaction (β = −0.532, *p* < 0.00). Hence, H2 is supported. Similarly, job satisfaction has a negative and significant impact on burnout (β = −0.136, *p* < 0.05), supporting the H3 hypothesis.

**Figure 2:**
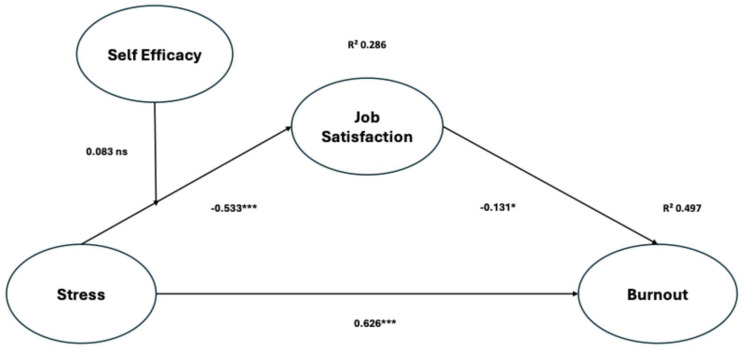
Hypothesized model and standardized estimate. *** *p* < 0.001; * *p* < 0.05; ns = not significant.

The mediation effect of job satisfaction in the relationship between occupational stress and burnout was found to be significantly positive (β = 0.107, *p* < 0.05) indicating a partial mediating effect. Thus, H4 was supported. Finally, the moderation effect of self-efficacy in the relationship between occupational stress and job satisfaction was not statistically significant (β = −0.083, *p* > 0. 05) indicating no moderating effect. Thus, H5 was rejected. Figure 2 depicts the results of the proposed structural model test.

## 6. Discussion

The present study explored the enduring and long-term associations among occupational stress, job satisfaction, self-efficacy, and burnout among nurses working at a government-affiliated university hospital in Saudi Arabia. The setting of this study is essential as public hospital staff experience numerous challenges, notably excessive workload, convoluted institutional structures within the organization, and enduring substantial stress. The present research contributes to the inadequate scholarship on occupational burnout within Middle Eastern healthcare systems, particularly within academic healthcare organizations.

The first hypothesis suggesting that work-related stress would significantly increase burnout has been confirmed (β = 0.626, *p* < 0.001). This corroborates findings from previous studies demonstrating that increased occupational stress contributes directly to higher rates of burnout in nurses [1,3]. The emotional exhaustion aspect of burnout tends to escalate under heavy workloads, time constraints, and the rigorous emotional demands of handling patient responsibilities; all these factors add to persistent stress leading to both physiological and emotional depletion that define burnout. The results appear to support research carried out in Saudi Arabia, which identified similar patterns among hospital nurses [31].

Hypothesis 2, which predicted that occupational stress negatively impacts an individual’s level of job satisfaction, was also confirmed (β = −0.533, *p* < 0.001). The evidence affirms that heightened stress adversely affects workplace atmosphere, leading to reduced motivation and lower levels of engagement, particularly among nursing professionals [42,44]. Chronic and unaddressed stressors not only erode the positive aspects of the job but also diminish the sense of reward, thereby worsening dissatisfaction. Within the Saudi context, compounded by situational factors such as protracted shifts, steep nurse-to-patient ratios, and minimal administrative aid, the evidence for reduced job satisfaction is well-documented [25].

Hypothesis 3, which states that job satisfaction negatively impacts burnout, was also supported (β = −0.131, *p* = 0.014). This aligns with global evidence showing that job satisfaction protects against emotional exhaustion and depersonalization [49,50]. Nurses who find a sense of purpose in their roles and feel valued by others tend to report lower levels of burnout, attributed to stronger psychological resilience and deeper professional commitment. This relationship has also been documented in international and local studies. El-Gilany and Amr reported that job satisfaction and burnout had an inverse relationship in nurses working in teaching hospitals in Egypt [67]. AbuAlRub in 2004 also reported these findings in Jordan, identifying job satisfaction as a major determinant of lower emotional exhaustion [38].

Within the Saudi context, Al-Otaibi and Al-Mutairi showed that greater satisfaction with one’s job led to decreased burnout symptoms [31]. Also, AlQahtani and Sayed in 2020 confirmed that job satisfaction was a major determinant for lower burnout and higher psychological well-being among nurses working in government hospitals [25].

Hypothesis 4 is confirmed with an effect of (β = 0.107, *p* = 0.004). This indicates that job satisfaction plays a partial mediating role in the association between occupational stress and burnout. It seems like a case that occupational stress leads to burnout through an indirect pathway where job satisfaction acts as a mediating factor and stress operates psychologically as emotional exhaustion along with depersonalization. These findings are in line with prior international studies supporting this mediation model [49,50,68,69]. Recent Saudi studies also provide additional evidence. A recent study by Shdaifat et al. (2023) demonstrated that job satisfaction served as a mediator in the link between occupational stress and burnout among nurses conducted within an academic hospital located in the Eastern region of Saudi Arabia [70]. Equally, Almetery et al. in 2023 identified a connection linking elevated work-related stress to reduced job satisfaction and increased levels of burnout [71]. The studies stress the importance of increasing job satisfaction as the primary strategy for reducing adverse consequences of stress and promoting better overall well-being among nursing staff. The fifth hypothesis, proposing that self-efficacy functions as a moderator between occupational stress and job satisfaction, was not supported by the data (β = −0.083, *p* = 0.334). Although self-efficacy is viewed as a personal resource that mitigates stress, the current findings are consistent with several studies indicating that the extent to which self-efficacy functions as a moderator may differ based on the contextual environment [72]. For instance, an Italy-based extensive investigation demonstrated the lack of significant self-efficacy moderation over stress and psychological well-being among nurses, explaining the result as a product of overwhelming institutional constraints [55]. Duffy et al. documented in 2019 comparable findings in U.S. healthcare settings [73].

Furthermore, the self-efficacy buffering effect may be more pronounced in individualistic or decentralized work environments, where personal initiative and decision-making have a greater impact. On the other hand, public hospitals as more hierarchical and protocol-driven contexts may curb the demonstration of self-efficacy, which dampens its buffering effects. This inconsistency highlights the context-sensitive models of burnout whereby personal resources like self-efficacy are juxtaposed with organizational boundaries, cultural norms, and styles of leadership. Indeed, researchers have observed this variability in various professional environments. Multiple studies have demonstrated that self-efficacy plays a notable moderating role in the connection between stress and burnout, particularly in professions characterized by greater autonomy, such as teaching and social work, where its influence tends to be positively amplified [50,53,74]. Locally, a Saudi study also concluded that there was no considerable self-efficacy moderating role in the impact of job stress among nurses, reinforcing the assumption that systemic factors seem to override individual coping capacities [10]. These studies emphasize the need to refrain from over-relying on enhanced personal psychological traits in dealing with burnout and instead focus on addressing systemic frameworks to improve worker dissatisfaction.

### 6.1. Implications

#### 6.1.1. Theoretical Implications

This research adds value to the theoretical literature through various impactful dimensions. To begin with, there exists a good number of studies focusing on the components of workplace stress, job contentment, belief in one’s capabilities, and emotional fatigue. Research of this nature is among the very first to try to bring many variables in one conceptual framework and provide new proof on the ways stress and organizational-level and individual-level factors interact to bring about burnout. Through an integrated perspective, one can understand the complicated relationship between work attributes such as workload and organizational and individual resources. Second, the research was conducted within a public academic hospital setting, which involves the integration of public healthcare services with teaching, supervision, and research. The unique combination of intensive clinical duties, teaching responsibilities, and academic demands in Saudi government university hospitals represents a high-pressure environment that has received limited attention in burnout literature. The focus of prior research has mostly been placed on private and public non-academic hospitals, primarily in Western countries. These gaps are addressed by the current study, which elucidated the burnout experiences of nurses working in one of the largest government university hospital in Saudi Arabia, thus expanding the institutional and cultural scope of burnout research. This specific context may tend to explain some of the findings, especially the restricted contribution of individual psychological resources such as self-efficacy, given that in such environments coping strategies tend to be minimal due to prevailing structural conditions.

Finally, the lack of significant moderation effect self-efficacy had on the stress–job satisfaction relationship poses interesting conceptual challenges concerning the restrictive role that personal resources may play in highly regulated organizational contexts. These findings highlight the need to reconsider the existing models of burnout and suggest that environmental rigidity could act as an intervening variable and suggest the need to refine or create new instruments to adequately reflect the interactions within these intricate work environments.

#### 6.1.2. Practical Implications

The results of this research hold notable significance for departments such as human resources, healthcare administrators, and policymakers. The demonstrated connection between occupational stress and burnout underscores the necessity of addressing ongoing workplace pressures. Reducing working hours, improving nurse–patient ratios, relieving layers of administration to alleviate the burden on nursing staff, and addressing other systemic factors may be applicable. The positive impact of work satisfaction and burnout suggests that an organizational climate may mitigate burnout impact. These goals can be accomplished through flexible shifts, recognition of contribution programs, promotion paths, and empowerment on morale-boosting team culture that enhances staff’s engagement. While self-efficacy did not show a direct moderating role on burnout, the absence of moderating role highlights a very critical insight: the healthcare system should not lean on the personal resilience or confidence of nurses to simply “pull through” chronic stress. In inflexible and high-demand situations, self-help strategies are unlikely to be effective without some form of systematic bolstering. Thus, there must be more comprehensive changes to shift the organizational need to improve workload, resources, and infrastructure.

These approaches align to provide the basis for transformative organizational change grounded in evidence that balances the needs of the individual and the institution, as well as reducing burnout while increasing the quality of care and above all, maintaining a safe environment for staff to work in.

### 6.2. Limitations, Recommendations, and Future Research

Although this study provides valuable contributions, some limitations must be acknowledged that will help with understanding the results and inform future studies. First, the investigation depended exclusively on participants’ self-assessments, which may have been subject to bias stemming from social desirability as well as subjective perceptions due to the common method bias. We encourage future research to integrate mixed methods with interviews, observational data, or even supervisor evaluations to refine the understanding of participants’ psychological experiences. Second, relying on a cross-sectional approach restricts the ability to capture how stress, burnout, job satisfaction, and self-efficacy evolve over time or to draw causal links among them [75]. Third, the sample was taken from a single governmental university hospital in Saudi Arabia, which is a hybrid public-serving and academic institution. This could potentially constrain the extent to which the results apply to broader settings beyond the current institutional context. Alenezi et al. have noted the need to include more institutions to capture contextual diversity regarding occupational stressors [76]. Fourth, while self-efficacy was directly linked to reduced burnout, the absence of a significant moderation effect could reflect limited measurement sensitivity rather than a lack of influence. In rigid and high-stakes contexts like public hospitals, self-efficacy measurement tends to overlook vital aspects of the healthcare workers’ psychological reality. As Shoji et al. pointed out, personal resources may be masked due to contextual demands [7]. Future researchers might focus on creating or modifying diagnostic instruments based on the actual experiences of frontline healthcare workers enduring persistent stress. Finally, this study does not incorporate other organizational concepts such as leadership style, team climate, or institutional justice. Instead, it focuses on four primary variables: occupational stress, burnout, job satisfaction, and self-efficacy. Such variables are known to influence staff well-being and engagement [14,77]. Considering such variables would enhance understanding of the burnout pathways in healthcare systems.

### 6.3. Conclusions

This study sheds some light on the relationships between workplace stress, job contentment, belief in one’s capabilities, and emotional fatigue in nursing, particularly in relation to high-stakes healthcare environments. The findings suggest systematically mitigating stressors and cultivating protective burnout resources at the individual, organizational, and systemic levels. The research further elucidated nurse well-being by establishing job satisfaction as an intermediary mechanism and self-efficacy as a core resilience factor, enriching the literature on nurses’ well-being in the context of a public academic hospital in Saudi Arabia. These findings strengthen the advocacy for comprehensive multifaceted frameworks alongside targeted responsive policies within burnout-affected workplaces. Research should strive to better understand multi-level and culturally specific dimensions of burnout to develop more precise and sustainable solutions.

## Figures and Tables

**Table 1:** Sample demographics (N = 245).

Demographic Profile	Category	Frequency	Percent (%)
Age	Under 25	54	22
	25–35	100	40.8
	36–44	71	29
	45 and above	20	8.2
Gender	Male	79	32.2
	Female	166	67.8
Nationality	Saudi	93	38
	Non-Saudi	152	62
Educational level	Diploma	141	57.6
	Bachelor’s degree	76	31
	Master’s degree	28	11.4
Experience	0–3 years	54	22
	4–8 years	25	10.2
	9–15 years	78	31.8
	15 years and above	88	35.9
Department	Orthopedics	11	4.5
	Dialysis	7	2.9
	Cardiac Catheterization unit	15	6.1
	Operating Room	21	8.6
	ER	46	18.8
	ICU	12	4.9
	OPD	15	6.1
	Ward—pediatrics	11	4.5
	Internal Medicine	41	16.7
	Ward—Surgery	54	22
	Oncology	7	2.9
	Cardiology	5	2

**Table 2:** Mean, standard deviation (SD), and correlation statistics.

Variables	Mean	Std. Deviation	OS	JS	SE	BU
OS	2.72	1.174	**0.976**			
JS	3.74	1.127	−0.532 **	**0.957**		
SE	2.54	0.755	0.016 ns	0.022 ns	**0.943**	
BU	3.67	1.794	0.696 **	−0.465 **	0.057 ns	**0.994**

Note: The value of Cronbach’s alpha is shown in bold in the matrix. ** *p* < 0.01 (two-tailed), and ns = non-significant. OS = occupational stress, JS = job satisfaction, SE = self-efficacy, and BU = burnout.

**Table 3:** Model fit of confirmatory factor analysis.

X^2^/df	*p*	GFI	CFI	TLI	SRMR	RMSEA	PCLOSE	AGFI
1.533	0.000	0.784	0.969	0.967	0.0276	0.047	0.880	0.760

Notes: CFI = comparative fit index; TLI = Tucker–Lewis’s index; SRMR = standardized root mean square residual; RMSEA = root mean square error of approximation; GFI = goodness of fit index; AGFI = adjusted goodness of fit index; PCLOSE = close fit with *p* value.

**Table 4:** Confirmatory factor analysis.

Construct	Items	Factor Loading	CR	AVE
Occupational stress	OS1	0.912	0.976	0.801
	OS10	0.889		
	OS2	0.924		
	OS3	0.883		
	OS4	0.921		
	OS5	0.881		
	OS6	0.883		
	OS7	0.898		
	OS8	0.897		
	OS9	0.861		
Job satisfaction	JS1	0.880	0.957	0.818
	JS2	0.914		
	JS3	0.935		
	JS4	0.857		
	JS5	0.934		
Self-efficacy	SE1	0.818	0.943	0.624
	SE10	0.777		
	SE2	0.772		
	SE3	0.763		
	SE4	0.841		
	SE5	0.817		
	SE6	0.744		
	SE7	0.793		
	SE8	0.768		
	SE9	0.799		
Burnout	PB1	0.932	0.932	0.873
	PB2	0.937		
	PB3	0.926		
	PB4	0.928		
	PB5	0.925		
	PB6	0.941		
	WB1	0.955		
	WB2	0.954		
	WB3	0.958		
	WB4	0.935		
	WB5	0.959		
	WB6	0.954		
	WB7	0.954		
	CB1	0.954		
	CB2	0.933		
	CB3	0.953		
	CB4	0.963		
	CB5	0.900		
	CB6	0.887		

Note: CR = composite reliability, AVE = average variance extracted. The factor loadings of all items are sufficiently high (>0.40) and statistically significant (*p* < 0.001).

**Table 5:** Structural model results. *** *p* < 0.001; ** *p* < 0.01; * *p* < 0.05.

Hypotheses	Relationships	Beta Coefficient	T Values	*p* Values	Decisions
	Direct effect				
H1	OS -> BU	0.626 ***	11.688	0.000	Significant
H2	OS -> JS	−0.533 ***	−9.856	0.000	Significant
H3	JS -> BU	−0.131 *	−2.451	0.014	Significant
	Mediation effect				
H4	OS -> JS -> BU	0.107 **	6.29	0.004	Significant
	Moderation effect				
H5	OS × SE -> JS	−0.083	−0.965	0.334	Non-significant

## Data Availability

Data sharing is not applicable. The data are not publicly available due to participants’ privacy.

## Abbreviations

OS	occupational stress
BO	burnout
SE	self-efficacy
JS	job satisfaction
CFA	confirmatory factor analysis
SEM	structural equation modeling
PSS	perceived stress scale
GSE	general self-efficacy scale
CSS	career satisfaction scale
(CBI)	Copenhagen Burnout Inventory

## References

[1] Maslach C., Leiter M.P. (2016). Burnout: The Cost of Caring. J. Occup. Health Psychol..

[2] World Health Organization (2019). Burn-Out an “Occupational Phenomenon”: International Classification of Diseases.

[3] Aiken L.H., Clarke S.P., Sloane D.M., Sochalski J., Silber J.H. (2002). Hospital nurse staffing and patient mortality, nurse burnout, and job dissatisfaction. JAMA.

[4] Freudenberger H.J. (1974). Staff burnout. J. Soc. Issues.

[5] Leiter M.P., Maslach C. (2004). Areas of worklife: A structured approach to organizational predictors of job burnout. Res. Occup. Stress Well Being.

[6] Xie Z., Wang A., Chen B. (2020). Nurse burnout and job satisfaction: A meta-analysis. Int. J. Nurs. Stud..

[7] Shoji K., Lesnierowska M., Smoktunowicz E., Benight C.C. (2016). Associations between job burnout and self-efficacy. Int. J. Stress Manag..

[8] Schwarzer R., Hallum S. (2008). Perceived self-efficacy as a protective factor in teachers’ burnout. Appl. Psychol. Int. Rev..

[9] Lim H., Lee S., Choi Y.J. (2022). Relationship between self-efficacy, burnout, and job performance among psychiatric nurses during COVID-19 pandemic. Medicina.

[10] Azemi S., Dianat I., Abdollahzade F., Bazazan A., Vahedian-Azimi A., Dehnavi Z.M. (2022). Self-efficacy as a mediator between work-related stress and mental health among hospital nurses in Iran. Work.

[11] Ibrahim H.A., Al-Khathami A. (2019). Work-related stress and burnout among nurses in Saudi public hospitals. J. Nurs. Manag..

[12] Judge T.A., Thoresen C.J., Bono J.E., Patton G.K. (2001). The job satisfaction–job performance relationship: A meta-analysis. Psychol. Bull..

[13] Garman A.N., Corrigan P.W., Morris S. (2002). Staff burnout and patient satisfaction: Evidence of relationships from a national survey. J. Healthc. Manag..

[14] Aiken L.H., Sermeus W., Van den Heede K., Sloane D.M. (2012). Nurse staffing and education and hospital mortality in nine European countries: A retrospective observational study. Lancet.

[15] Estryn-Béhar M., Van der Heijden B.I., Oginska H., Camerino D., Le Nezet O., Conway P.M., Fry C., Hasselhorn H.-M. (2007). NEXT Study Group. The impact of social work environment, teamwork characteristics, burnout, and personal factors upon intent to leave among European nurses. Med. Care.

[16] Al Mutair A.S., Al Obaid R.S., Al Mutairi N.S. (2021). The effect of work-related stress on job satisfaction and burnout among Saudi nurses. Saudi J. Nurs. Health Sci..

[17] AlHadi A.N., Almutlaq M.I., Almohawes M.K., Shadid A.M., Alangari A.A. (2022). Prevalence and treatment preference of burnout, depression, and anxiety among mental health professionals in Saudi Arabia. J. Nat. Sci. Med..

[18] Halabi J., Abu-Snieneh H. (2021). Predictors of burnout among nurses in Jordan: The impact of workload and organizational support. J. Nurs. Res..

[19] Gomez-Urquiza J.L., De la Fuente-Solana E.I., Albendín-García L., Vargas-Pecino C., Ortega-Campos E., Cañadas-De la Fuente G.A. (2017). Prevalence of burnout syndrome in emergency nurses: A meta-analysis. Crit. Care Nurs..

[20] Sami W. (2023). Burnout among nurses in Cairo’s public hospitals. Egypt J. Nurs..

[21] Ahmed H. (2021). Self-efficacy and occupational stress in Egyptian nurses. Int. J. Nurs. Stud..

[22] Nour Eldin A. (2022). The role of job satisfaction in reducing burnout in Egyptian healthcare. Middle East J. Nurs..

[23] Sami W., Al-Maqbali M., Alghamdi A. (2023). Occupational stress, job satisfaction and intention to leave among nurses working in critical care settings in Jordan: A cross-sectional study. J. Nurs. Manag..

[24] Alfuqaha O.A., Alkawareek M.Y., Alsharah H.S. (2019). Self-evaluation and professional status as predictors of burnout among nurses in Jordan. PLoS ONE.

[25] AlQahtani M.M., Sayed A.A. (2020). Job satisfaction among Saudi nurses: A cross-sectional study. J. Nurs. Manag..

[26] Alharbi J., Wilson R., Woods C. (2016). Self-efficacy, job stress, and burnout among nurses in Saudi Arabia. Int. J. Nurs. Pract..

[27] Al-Haroon H.I., Al-Qahtani M.F. (2020). The demographic predictors of job satisfaction among the nurses of a major public hospital in KSA. J. Taibah Univ. Med. Sci..

[28] Kim H., Ji J., Kao D. (2011). Burnout and physical health among social workers: A three-year longitudinal study. Soc. Work.

[29] Kristensen T.S., Borritz M., Villadsen E., Christensen K.B. (2005). The Copenhagen Burnout Inventory: A new tool for the assessment of burnout. Work. Stress.

[30] Adriaenssens J., De Gucht V., Maes S. (2015). Determinants and prevalence of burnout in emergency nurses: A systematic review and meta-analysis. J. Adv. Nurs..

[31] Al-Otaibi H., Al-Mutairi S. (2021). Occupational stress and burnout among nurses in Saudi Arabia: A cross-sectional study. Saudi Med. J..

[32] Schaufeli W.B., Enzmann D. (1998). The Burnout Companion to Study and Practice: A Critical Analysis.

[33] Laschinger H.K.S., Finegan J., Wilk P. (2011). Situational and dispositional influences on nurses’ workplace well-being: A time-lagged analysis. J. Nurs. Manag..

[34] Leiter M.P., Maslach C. (2017). Burnout and engagement: Contributions to a new vision. Burn. Res..

[35] Brough P., Driscoll O. (2010). Work-family conflict and stress in nurses. J. Occup. Health Psychol..

[36] Khamisa N., Oldenburg B., Peltzer K., Ilic D. (2015). Work-related stress, burnout, job satisfaction, and general health of nurses. Int. J. Environ. Res. Public Health.

[37] Leiter M.P., Bakker A.B., Maslach C. (2014). Burnout at Work: A Psychological Perspective.

[38] AbuAlRub R.F. (2004). Job stress, job performance, and social support among hospital nurses. J. Nurs. Sch..

[39] Al-Omari A., Al Mutair A., Shamsan A., Al Mutairi A. (2020). Predicting burnout factors among healthcare providers at private hospitals in Saudi Arabia and United Arab Emirates: A cross-sectional study. Appl. Sci..

[40] Clinton M., Bou-Karroum K., Doumit M.A., Richa N., Alameddine M. (2022). Determining levels of nurse burnout during the COVID-19 pandemic and Lebanon’s political and financial collapse. BMC Nurs..

[41] Khalil H.A., Aziz A.R. (2020). Occupational burnout in Iraqi nurses: The role of stressors and psychological support in war zones. Middle East J. Psychiatry.

[42] Browning E.D. (2019). Moral distress and psychological empowerment in critical care nurses caring for patients with COVID-19. Dimens. Crit. Care Nurs..

[43] Moss M., Good V.S., Gozal D., Kleinpell R., Sessler C.N. (2016). A critical care societies collaborative statement: Burnout syndrome in critical care health-care professionals. Am. J. Respir. Crit. Care Med..

[44] Pappa S., Ntella V., Giannakas T., Giannakoulis V.G., Papoutsi E., Katsaounou P. (2020). Prevalence of depression, anxiety, and insomnia among healthcare workers during the COVID-19 pandemic: A systematic review and meta-analysis. Brain Behav. Immun..

[45] Arias-Ulloa C.A., Huamani C., Calizaya-Milla Y.E. (2023). Psychological distress and its association with burnout and work engagement in healthcare workers during the COVID-19 pandemic. Int. J. Ment. Health Nurs..

[46] Frese M. (1985). Stress at work and psychosomatic complaints: A causal interpretation. J. Appl. Psychol..

[47] Parkes K.R. (1982). Occupational stress among student nurses: A natural experiment. J. Appl. Psychol..

[48] Kim S.Y., Park H.J., Lee H. (2023). Longitudinal effects of occupational stress on job satisfaction and career retention in Korean nurses: A 2023 prospective study. Int. J. Nurs. Stud..

[49] Theorell T., Hammarström A., Aronsson G., Träskman Bendz L., Grape T., Hogstedt C., Marteinsdottir I., Skoog I., Hall C. (2015). A systematic review including meta-analysis of work environment and depressive symptoms. BMC Public Health.

[50] Häusser J.A., Mojzisch A., Niesel M., Schulz-Hardt S. (2010). Ten years on: A review of recent research on the Job Demand–Control (Support) model and psychological well-being. Work. Stress.

[51] Zhang Y., Liu H., Zhang L., Wang Y., Bai M., Wang Y. (2017). Workplace violence against nurses: A cross-sectional study. Int. J. Nurs. Stud..

[52] Zhang Y., Zhang Y., Wang J., Wang P. (2017). The relationship between workplace violence and job satisfaction among nurses in China: The mediating role of burnout. J. Nurs. Manag..

[53] Dollard M.F., Bailey T.S., McLinton S.S., Richards P.A. (2015). Psychosocial safety climate, psychosocial and physical risk factors in the workplace. Health Promot. Int..

[54] Rafiei S., Azizi S.M., Sabzevari S., Zarea K. (2024). The moderating role of self-efficacy in the relationship between occupational stress and mental health in nurses: A cross-sectional study. J. Psychiatr. Ment. Health Nurs..

[55] Pisanti R., van der Doef M., Maes S., Meier L.L., Lazzari D., Violani C. (2015). Occupational coping self-efficacy explains distress and well-being in nurses beyond psychosocial job characteristics. Front. Psychol..

[56] Pisanti R., Lombardo C., Lucidi F., Lazzari D., Bertini M. (2008). Development and validation of a brief Occupational Coping Self-Efficacy Questionnaire (OCSE) for stress-related job conditions. J. Psychosom. Res..

[57] Cabrera-Aguilar M., Sánchez-García M., López-González L., Hidalgo-Rasmussen C. (2023). Self-efficacy as a moderator between job stress and work engagement among nurses: A cross-sectional study. Int. J. Environ. Res. Public Health.

[58] Brislin R.W. (1970). Back-translation for cross-cultural research. J. Cross-Cult. Psychol..

[59] Cohen S., Kamarck T., Mermelstein R. (1983). A global measure of perceived stress. J. Health Soc. Behav..

[60] Chaaya M., Osman H., Naassan G., Mahfoud Z. (2010). Validation of the Arabic version of the Cohen Perceived Stress Scale (PSS-10) among pregnant and postpartum women. BMC Psychiatry.

[61] Schwarzer R., Jerusalem M., Weinman J., Wright S., Johnston M. (1995). Generalized Self-Efficacy Scale. Measures in Health Psychology: A User’s Portfolio; Causal and Control Beliefs.

[62] Dardas L.A., Silva S.G., Smoski M.J., Noonan D., Simmons L.A. (2017). The Arabic version of the General Self-Efficacy Scale: Psychometric evaluation in Saudi Arabia. J. Nurs. Meas..

[63] Greenhaus J.H., Parasuraman S., Wormley W.M. (1990). Effects of race on organizational experiences, job performance evaluations, and career outcomes. Acad. Manag. J..

[64] Al-Fadhel M., Khalid K. (2021). Career satisfaction scale: Translation and validation of an Arabic version among Kuwaiti employees. Int. J. Bus. Soc. Sci..

[65] Hair J.F., Black W.C., Babin B.J., Anderson R.E. (2010). Multivariate Data Analysis.

[66] Fornell C., Larcker D.F. (1981). Evaluating structural equation models with unobservable variables and measurement error. J. Mark. Res..

[67] El-Gilany A.H., Amr M. (2010). Job satisfaction and burnout among nurses in Al-Hassa, Saudi Arabia and Alexandria, Egypt. J. Egypt Public Health Assoc..

[68] Bakker A.B., Demerouti E., Verbeke W. (2004). Using the Job Demands-Resources model to predict burnout and performance. Hum. Resour. Manag..

[69] Van der Heijden B.I.J.M., Mulder R.H., König C., Anselmann V. (2019). Toward a mediation model for nurses’ well-being and psychological distress effects of quality of leadership and social support at work. Medicine.

[70] Shdaifat E.A., Alquwez N., Almazan J.U., Tork H.M., Alsolami F., Tumala R.B. (2023). The mediating role of job satisfaction between occupational stress and burnout among nurses in a teaching hospital in Saudi Arabia. J. Taibah Univ. Med. Sci..

[71] Almetery A., Alghamdi A., Shalabi R., Alshehri R. (2023). Exploring work-related stress and burnout among nurses: The role of job satisfaction. Saudi J. Health Sci..

[72] Bandura A. (1997). Self-Efficacy: The Exercise of Control.

[73] Duffy R.D., Autin K.L., Douglass R.P. (2019). Examining self-efficacy as a moderator of the relation between work stressors and work meaning. J. Career Assess..

[74] Zhang Y., Zhang Y., Zhang Y., Su H. (2017). The impact of workplace violence on job satisfaction and burnout among nurses: A cross-sectional study. Int. J. Nurs. Stud..

[75] Leiter M.P., Maslach C. (2005). Banishing Burnout: Six Strategies for Improving Your Relationship with Work.

[76] Alenezi A.M., Alshammari F.S., Alotaibi S.T. (2022). Occupational stress and job satisfaction among healthcare providers in various healthcare settings: A comparative study. Saudi Med. J..

[77] Laschinger H.K.S., Wong C.A., Greco P. (2001). The impact of staff nurse empowerment on person–job fit and work engagement/burnout. Nurs. Adm. Q..

